# Awareness of episodic memory and meta-cognitive profiles: associations with cerebrospinal fluid biomarkers at the preclinical stage of the Alzheimer’s continuum

**DOI:** 10.3389/fnagi.2024.1394460

**Published:** 2024-05-30

**Authors:** David López-Martos, Marc Suárez-Calvet, Marta Milà-Alomà, Juan Domingo Gispert, Carolina Minguillon, Clara Quijano-Rubio, Gwendlyn Kollmorgen, Henrik Zetterberg, Kaj Blennow, Oriol Grau-Rivera, Gonzalo Sánchez-Benavides

**Affiliations:** ^1^Barcelonaβeta Brain Research Center (BBRC), Pasqual Maragall Foundation, Barcelona, Spain; ^2^Hospital del Mar Research Institute (IMIM), Barcelona, Spain; ^3^Centro de Investigación Biomédica en Red de Fragilidad y Envejecimiento Saludable, Instituto de Salud Carlos III, Madrid, Spain; ^4^Servei de Neurologia, Hospital del Mar, Barcelona, Spain; ^5^Department of Veterans Affairs Medical Center, Northern California Institute for Research and Education (NCIRE), San Francisco, CA, United States; ^6^Department of Radiology, University of California San Francisco, San Francisco, CA, United States; ^7^Centro de Investigación Biomédica en Red Bioingeniería, Biomateriales y Nanomedicina, Madrid, Spain; ^8^Roche Diagnostics International Ltd, Rotkreutz, Switzerland; ^9^Roche Diagnostics GmbH, Penzberg, Germany; ^10^Department of Psychiatry and Neurochemistry, Institute of Neuroscience and Physiology, University of Gothenburg, Mölndal, Sweden; ^11^Clinical Neurochemistry Laboratory, Sahlgrenska University Hospital, Mölndal, Sweden; ^12^Department of Neurodegenerative Disease, UCL Institute of Neurology, London, United Kingdom; ^13^UK Dementia Research Institute at UCL, London, United Kingdom; ^14^Hong Kong Center for Neurodegenerative Diseases, Clear Water Bay, Hong Kong, Hong Kong SAR, China; ^15^Wisconsin Alzheimer’s Disease Research Center, University of Wisconsin School of Medicine and Public Health, University of Wisconsin-Madison, Madison, WI, United States; ^16^Paris Brain Institute, ICM, Pitié-Salpêtrière Hospital, Sorbonne University, Paris, France; ^17^Neurodegenerative Disorder Research Center, Division of Life Sciences and Medicine, and Department of Neurology, Institute on Aging and Brain Disorders, University of Science and Technology of China and First Affiliated Hospital of USTC, Hefei, China

**Keywords:** Alzheimer’s disease, preclinical, awareness, episodic memory, biomarkers

## Abstract

**Introduction:**

The lack of cognitive awareness, anosognosia, is a clinical deficit in Alzheimer’s disease (AD) dementia. However, an increased awareness of cognitive function, hypernosognosia, may serve as a marker in the preclinical stage. Subjective cognitive decline (SCD) might correspond to the initial symptom in the dynamic trajectory of awareness, but SCD might be absent along with low awareness of actual cognitive performance in the preclinical stage. We hypothesized that distinct meta-cognitive profiles, both hypernosognosia and anosognosia, might be identified in preclinical-AD. This research evaluated the association between cerebrospinal fluid (CSF) AD biomarkers and the awareness of episodic memory, further exploring dyadic (participant-partner) SCD reports, in the preclinical Alzheimer’s continuum.

**Methods:**

We analyzed 314 cognitively unimpaired (CU) middle-aged individuals (mean age: 60, SD: 4) from the ALFA+ cohort study. Episodic memory was evaluated with the delayed recall from the Memory Binding Test (MBT). Awareness of episodic memory, meta-memory, was defined as the normalized discrepancy between objective and subjective performance. SCD was defined using self-report, and dyadic SCD profiles incorporated the study partner’s report using parallel SCD-Questionnaires. The relationship between CSF Aβ42/40 and CSF p-tau181 with meta-memory was evaluated with multivariable regression models. The role of SCD and the dyadic contingency was explored with the corresponding stratified analysis.

**Results:**

CSF Aβ42/40 was non-linearly associated with meta-memory, showing an increased awareness up to Aβ-positivity and a decreased awareness beyond this threshold. In the non-SCD subset, the non-linear association between CSF Aβ42/40 and meta-memory persisted. In the SCD subset, higher Aβ-pathology was linearly associated with increased awareness. Individuals presenting only study partner’s SCD, defined as unaware decliners, exhibited higher levels of CSF p-tau181 correlated with lower meta-memory performance.

**Discussion:**

These results suggested that distinct meta-cognitive profiles can be identified in preclinical-AD. While most individuals might experience an increased awareness associated with the entrance in the AD continuum, hypernosognosia, some might be already losing insight and stepping into the anosognosic trajectory. This research reinforced that an early anosognosic profile, although at increased risk of AD-related decline, might be currently overlooked considering actual diagnostic criteria, and therefore its medical attention delayed.

## 1 Introduction

Alzheimer’s disease (AD) is a progressive neurodegenerative disorder, causing cognitive, behavioral, and psychiatric symptoms that involve functional impairment in the stage of dementia. The episodic memory system is the central node of disruption in the cognitive architecture of Alzheimer’s (Grober et al., 1987, 2008, 2023). One of the most striking symptoms of Alzheimer’s is anosognosia, a clinical deficit consisting in the patient’s unawareness of the neurological disease, showing lack of appraisal in cognitive, behavioral, and/or functional capacity (Starkstein et al., 2006; Starkstein, 2014). The cognitive neuropsychology of anosognosia in amnestic patients has contributed substantially to delineate the phenomenology of AD dementia, suggesting a selective disconnection between the modules of memory and awareness (McGlynn and Schacter, 1989; Schacter, 1990, 1992). Anosognosia, particularly referring here to the awareness of cognitive deficits in AD patients, might arise from a disruption in the meta-cognitive system. Meta-cognition supports a crucial role in self-referential processing and awareness of cognitive function, considered as a high order cognitive function involving the control and monitoring of thoughts, cognitive processes, and mental states (Sunderaraman and Cosentino, 2017).

Alzheimer’s neuropathology, consistent in abnormal deposition of amyloid-β (Aβ) plaques and tau tangles in the brain, defines the biological presence of the disease. These proteins begin to accumulate decades before the explicit occurrence of the first clinical symptoms (preclinical-AD). Along with the underlying neuropathological progression, the overt manifestation of AD gradually advances from healthy cognition to mild cognitive impairment (MCI), and ultimately, AD dementia. The status of amyloid-β (A), tau (T), and non-specific neurodegeneration ([N]) biomarkers are used to describe in a research framework the AT(N) profiles along the Alzheimer’s continuum (Jack et al., 2018). The current clinical criteria from the National Institute on Aging and the Alzheimer’s association (NIA-AA) characterize the transitional stage between healthy cognition and MCI by considering longitudinal cognitive assessment and/or subjective cognitive complaints (Jack et al., 2018). Currently, a critical point in the field is to uncover the biological, cognitive, and behavioral underpinnings of the disease’s trajectory, allowing early detection and intervention at the preclinical stage of the Alzheimer’s continuum.

Subjective Cognitive Decline (SCD) is defined as the self-perception of having experienced a change from the previous/expected cognitive capacity despite a clinically normal objective performance, directly related to middle aged and older population presenting concerns related to their own cognitive health. SCD is considered a risk factor for MCI, and it may represent one of the initial symptomatic manifestations of AD (Jessen et al., 2014). Nevertheless, subtle cognitive decline may or may not be evident to the individuals themselves or their close family members. Moreover, cognitive complaints can occur independently of AD pathological changes, and many other factors might lead to SCD, ranging from healthy aging to different clinical disorders (Jessen et al., 2014, 2020). Therefore, the assessment of SCD might result non-specific for AD, and it is currently recognized that that most individuals with SCD will not progress to MCI. Importantly, in the context of self-perception of cognitive function, it has been shown that subclinical levels of anxiety and depression are associated with SCD (Hill et al., 2016; Jenkins et al., 2021), but these symptoms might be: i) a plausible cause of SCD, ii) manifestation of underlying Aβ pathology, or iii) consequence of experiencing SCD (Jessen et al., 2020).

Subtle psychiatric symptoms have been linked with AD biomarkers and the risk of progression in CU individuals (Donovan et al., 2018; Krell-Roesch et al., 2018; Hanseeuw et al., 2020a; Lewis et al., 2022). Therefore, SCD and affective symptoms may interact, and their co-occurrence has already been linked with cognitive decline (Liew, 2020). The Subjective Cognitive Decline Initiative (SCD-I) working group suggested that subthreshold symptoms of anxiety and depression should be accounted in models of preclinical-AD, since these affective characteristics might be manifestations of AD-pathology as well (Jessen et al., 2014). Beyond these considerations, SCD criteria provides important and valuable insights into the early detection of AD pathology and the risk of clinical progression (Jessen et al., 2014, 2020). More recently, measurements of the awareness of cognitive function have been suggested to be a more specific marker than self-reported SCD at the preclinical stage of Alzheimer’s (Cacciamani et al., 2021).

In the context of clinical research in AD, cognitive awareness has been mainly measured through clinician evaluation, participant-partner discrepancy in parallel questionnaires of SCD, and/or objective-subjective discrepancy in neuropsychological performance (Starkstein, 2014). Awareness is commonly altered in MCI and AD dementia patients, with anosognosia reaching up to 80% of estimated prevalence in AD patients (Starkstein, 2014). Anosognosia is a notorious symptom that is associated with the severity of AD dementia, it relates with the patient’s involvement in dangerous behaviors, psychiatric and behavioral problems, and with increased caregiver burden (Horning et al., 2014; Starkstein, 2014). An impaired meta-cognition in AD due to anosognosia might come along with a widespread range of clinical implications, for both patients and their families. Improving our understanding of the cognitive, neuronal, and pathological underpinnings of anosognosia would enhance preclinical stage identification, informing about risk of progression and more precise therapeutic interventions. At the present, however, it is unclear how to delineate the spectrum of distinct subclinical alterations in the awareness of cognitive function present in preclinical-AD.

Cacciamani et al. (2017) presented the first study considering the specificity of awareness prior to the onset of clinical symptoms. They defined awareness of cognitive decline (ACD) using the participant-partner discrepancy in SCD questionnaires, characterizing participants as having low (participant complaints < partner complaints) or high ACD (participant complaints > partner complaints). This study showed that low ACD was associated with greater Aβ load and reduced cortical metabolism in comparison to individuals with high ACD. These insights were accompanied with the note that SCD measures, which involved only the participant’s self-reported SCD, without incorporating the study partner’s SCD, failed to show any consistent association with AD biomarkers. Vannini et al. (2017a) provided a chronological model of awareness across the preclinical and prodromal stages of AD. Considering here the discrepancy between objective and subjective memory performance, they showed that AD dynamically impacts awareness of episodic memory function. Their results indicated that in CU individuals, Aβ-pathology was associated with increased awareness of cognitive function, hypernosognosia, while Aβ-pathology in MCI patients was associated with reduced awareness of cognitive function, anosognosia, indicating a switch in the trajectory of meta-cognition in AD.

We have carried on a magnetic resonance imaging (MRI) study in our group, Sánchez-Benavides et al. (2018a), classifying CU individuals with the participant-partner SCD discrepancy as: unaware decliners (presenting only study partner’s SCD), SCD (self-reported SCD regardless of the study partner’s SCD), and controls (non-SCD and study partner’s non-SCD; neither participant nor study partner reported SCD). We found that unaware decliners showed lower memory performance, along with increased grey matter (GM) volume in medial frontal and insular regions in comparison to controls. The presence of SCD reported by the study partner, regardless of whether the participant themselves reported SCD, was as a significant predictor of lower hippocampal GM volume. These results were discussed in the context of non-linear changes preceding loss of GM volume in self-referential processing brain areas prior to clinical onset, and further suggested that observations made by a study partner regarding cognitive changes are a relevant piece of clinical information holding diagnostic value, independently of the individual’s self-awareness of cognitive decline. Following the literature of non-linear changes at the preclinical stage, Gagliardi et al. (2020) conducted research on awareness in the context of SCD, indicating that Aβ-pathology was non-linearly associated with awareness of episodic memory (objective-subjective discrepancy), showing hypernosognosia up to the threshold of Aβ-positivity, and anosognosia beyond the threshold of the Alzheimer’s pathological change. These results were discussed considering that frontal amyloid deposition might interfere with regions/networks of awareness and cognitive control, suggesting that the non-linear impact on awareness thus might relate to the advancement of Aβ-pathology.

Some longitudinal studies have already addressed the trajectory of awareness in the AD continuum. Hanseeuw et al. (2020b) showed that Aβ-pathology was associated with progressive lack of awareness, as defined with the participant-partner discrepancy. This study indicated that, regardless of clinical progression, the overall tendency in CU participants was an increased awareness. Crucially, considering just participants with clinical progression (i.e., CU to MCI, or MCI to dementia) the clinical status was predicted only with low, but not high awareness. The timeline showed that an increase in awareness was observed 1.6 years before MCI diagnosis, with awareness declining until symptom onset. In MCI patients, awareness was initially low and continued to decrease reaching anosognosia 3.2 years before dementia diagnosis. Cacciamani et al. (2020) described 3 longitudinal trends of ACD evolution (heightened, stable, and decline) in relationship to Aβ-pathology in a SCD cohort, showing in consistency, that progressive lack of awareness, but not persistence of cognitive complaints, was associated with greater Aβ-accumulation. A recent longitudinal study explored these distinct subclinical manifestations considering the awareness of cognitive function in relation to risk of progression in CU individuals, showing again that decreased, but not increased awareness, was associated with higher risk of clinical progression (Mimmack et al., 2023).

Therefore, distinct studies have suggested that prior to clinical onset in the AD continuum, CU individuals might experience a heightened awareness of cognitive function associated with underlying AD-progression, while other studies have suggested that lack of awareness is associated with similar characteristics and clinical progression. Although non-linear changes across preclinical AD have been already proposed, the heterogeneous levels of awareness shown by different individuals in relationship with AD neuropathology is a phenomenon not yet fully understood. Particularly, it is still unclear whether increased or decreased awareness of cognitive function characterizes best preclinical-AD. One the one side, SCD might represent the initial symptom of AD dementia, but on the other side, SCD might be not related to AD, or absent in preclinical stage for individuals progressing to MCI, as well as it can be absent in individuals progressing from MCI to AD (Mitchell et al., 2014). Based on the latest research, seems reasonable that distinct meta-cognitive profiles might emerge in preclinical-AD. In the present research, we hypothesized that, at very early stages in the preclinical Alzheimer’s continuum, there may be two distinct types of metacognitive profiles at risk of AD-related impairment:

(I) Hypernosognosia: Individuals with a sub-estimation of actual memory performance, showing an increased awareness in relationship to AD neuropathology.
(II) Anosognosia: Individuals with an over-estimation of actual memory performance, showing a decreased awareness in relationship to AD neuropathology.

The main objective of the present research was to test this hypothesis in the ALFA+ cohort study by evaluating the association of Cerebrospinal Fluid (CSF) Aβ and Tau biomarkers with awareness of episodic memory. Additionally, the role of self-reported and dyadic (participant-partner) SCD was further explored in these associations at the preclinical stage of the Alzheimer’s continuum.

## 2 Materials and methods

### 2.1 Participant characteristics

The present research was performed in the ALFA+ cohort study, a longitudinal study nested to the ALFA study (Alzheimer’s and Families) (Molinuevo et al., 2016). The initial ALFA study included 2,743 middle-aged CU individuals with a high proportion of AD patients’ offspring (47.4%) and apolipoprotein E (*APOE*) ε4 carriers (34.8%). The nested ALFA+ study included 450 participants selected by their specific AD risk profile (AD parental history, and *APOE*-ε4 status) (Molinuevo et al., 2016). A detailed phenotyping of the participants, aside from a clinical, cognitive, and lifestyle characterization, involved blood and CSF sample collection for biomarker determination, as well as magnetic resonance imaging (MRI) and positron emission tomography (PET) acquisition.

The ALFA+ inclusion criteria were: (1) individuals who had previously participated in the ALFA study; (2) age between 45 and 65 years at the inclusion in ALFA; and (3) long-term commitment to the study: inclusion and follow-up visits and agreement to undergo all tests and study procedures (MRI, PET, and lumbar puncture). ALFA+ exclusion criteria were: (1) cognitive impairment (Clinical Dementia Rating [CDR] > 0, Mini-Mental State Examination (MMSE) < 27 or semantic fluency < 12); (2) any systemic illness or unstable medical condition that could lead to difficulty complying with the protocol; (3) any contraindication to any test or procedure; and (4) a family history of monogenic AD. In the present study, we included 314 individuals with complete CSF biomarker measurements, and cognitive data.

The ALFA+ study (ALFA-FPM-0311) was approved by the independent ethics committee ‘Parc de Salut Mar’, Barcelona, and registered at Clinicaltrials.gov (identifier: NCT02485730). All participants signed the study’s informed consent form also approved by the independent ethics committee ‘Parc de Salut Mar’, Barcelona.

### 2.2 Sample collection and biomarker measurements

Cerebrospinal fluid sample collection and processing followed standard procedures (Teunissen et al., 2014), which have been previously described (Milà-Alomà et al., 2020). CSF Aβ40 and Aβ42 were measured with the exploratory NeuroToolKit, a panel of robust prototype immunoassays (Roche Diagnostics International Ltd, Rotkreuz, Switzerland), on a cobas^®^ e 601 module. CSF p-tau181 and t-tau (both corresponding to the mid-region domain of tau protein) were measured using the electrochemiluminescence Elecsys^®^ Phospho-Tau (181P) CSF and Elecsys Total-Tau CSF immunoassays, respectively, on a fully automated cobas e 601 module (Roche Diagnostics International Ltd, Rotkreuz, Switzerland). All CSF biomarker measurements were determined at the Clinical Neurochemistry Laboratory at the University of Gothenburg, Sweden.

### 2.3 AT classification system and biomarker profiles

Following the NIA-AA research criteria (Jack et al., 2018) to define the AD neuropathologic processes, we used the AT system to classify each biomarker status. AT groups were defined using CSF levels with the cut-off of 0.071 of the CSF Aβ42/40 ratio for A status, and the cut-off of 24 pg/mL of CSF p-tau181 for T status (Milà-Alomà et al., 2020). We excluded from the analysis 12 participants classified with suspected non-Alzheimer’s pathology (A-T+) and included only those with normal biomarker levels (A-T-) or already in the Alzheimer’s continuum (A+ T-, A+T+) (Jack et al., 2018).

### 2.4 Neuropsychological assessment

The Memory Binding Test (MBT) (Buschke, 2013) Spanish version (Gramunt et al., 2016) was used to assess verbal episodic memory through associative learning. The variables used in the present research to assess objective performance considered the two delayed recall measurements (30 min after learning): the total delayed free recall (0–32), and the total delayed paired recall (0–32). Participants were instructed to self-rate their overall performance in a single score ranging from 0 (worst possible performance) to 100 (best possible performance) after completing both delayed recall tasks. Objective episodic memory performance resulted from the arithmetic mean of the delayed recall (considering the total delayed free recall and the total delayed paired recall). Specifically, the total number of words recalled, both at free and cued delayed recall, was divided by the maximum possible score, and next multiplied by 100, thus obtaining a percentage. This way, objective performance was normalized to a scale from 0 to 100, matching post-diction metrics. A multivariable regression model was used to extract objective and subjective residual memory scores adjusted for demographic effects (sex, age, and education). Considering the full sample of participants, objective and subjective residual scores were standardized, dividing the residuals of each regression model by their estimated standard deviation. Meta-memory standardized residuals were computed subtracting subjective from objective standardized residuals, with positive scores indicating sub-estimation and negative scores indicating over-estimation of actual episodic memory performance.

The Subjective Cognitive Decline Questionnaire (SCD-Q) (Rami et al., 2014) was used as criteria to characterize the participant’s and study partner’s perception of SCD with parallel questionnaires, My-Cognition and Their-Cognition, respectively. The SCD-Q is a validated tool devised to detect and quantify the perceived subjective cognitive decline, comprising the same set of questions for the subject and the informant. The SCD-Q contains 3 initial “yes/no” questions followed by 24 items, inquiring about the presence or absence of difficulties in cognitive-related activities (for each participant and informant parallel versions). In line with previous studies, the presence/absence of subjective cognitive decline (SCD/non-SCD), as well as the mirror for the study partner (study partner’s SCD/study partner’s non-SCD), was defined with “yes/no” to this initial general question: “*Do you perceive memory or cognitive difficulties?*” for the participant, and “*Do you perceive he/she has cognitive or memory difficulties?*” for the study partner (Sánchez-Benavides et al., 2018a,b, 2021; Akinci et al., 2022).

The Hospital Anxiety and Depression Scale (HADS) (Zigmond and Snaith, 1983) was used to assess affective symptoms. The variables used in the present research were the anxiety (0–21) and depression (0–21) sub-scores from the HADS.

### 2.5 Statistical analyses

The analyses were divided into three main blocks. First, main analyses in the whole sample evaluating associations between meta-memory and CSF biomarkers. Second, stratified analyses by SCD status, using two models with the same predictors but in distinct subsets of the sample (non-SCD and SCD). Third, stratified analyses once the study partner’s SCD report was incorporated, combining it with the self-reported SCD measurements previously explored. Here, we considered to focus only on the subset characterized with presence of study partner’s SCD: within this subset, the sample was further stratified considering the self-reported SCD. Thus, there were “unaware decliners” (only the study partner presented SCD), and “aware decliners” (self-reported SCD confirmed by the study partner).

All CSF measurements and meta-memory scores were treated as continuous variables and tested for normality using the Kolmogorov-Smirnov normality test and visual inspection of histograms. In line with previous work, extreme values were excluded for CSF biomarkers (outlier threshold of 3 times the Interquartile Range [IQR]) (Milà-Alomà et al., 2020). The CSF Aβ42/40 ratio followed a normal distribution, but the CSF p-tau181 was not normally distributed and was log10-transformed. Meta-memory scores followed a normal distribution. In the main multivariable regression model, we evaluated the relationship between CSF biomarkers and meta-memory standardized residual scores adjusted for demographics. We used CSF Aβ42/40, and CSF p-tau181, incorporating a quadratic term for CSF Aβ42/40, and the interaction between CSF p-tau181 and the quadratic term for CSF Aβ42/40.

The inclusion of the quadratic term for CSF Aβ42/40 has been justified with theoretical and statistical considerations. Previous research suggested a switch in the trajectory of awareness across preclinical-AD (Vannini et al., 2017a). To the best of our knowledge, only one study to date have explicitly described a non-linear relationship between the continuous levels of Aβ pathology and the awareness of cognitive function in preclinical-AD (Gagliardi et al., 2020). The early impact of Aβ has been associated with awareness of memory performance due to disruption in frontal brain regions supporting cognitive control. While tau is more associated than Aβ with clinical symptoms at later disease stages, both pathologies have synergistic effects (Busche and Hyman, 2020). Yet, there is no literature suggesting that tau (presumably impacting medial temporal lobe) might induce non-linear changes in cognitive function prior to clinical onset.

The assumptions of the multivariable regression models were statistically tested for normality, homoscedasticity, and independence of residuals. Standardized β coefficients with 95% confidence interval (CI) and corresponding *p* values were reported. A descriptive correlation, instead of regression, was used to analyze associations between meta-memory and CSF biomarkers only after incorporating study partner’s SCD data, since the low sample size restricted the use of inferential statistics. For these analyses, Spearman correlation coefficients with corresponding *p* values were reported. For all statistical models, including regression and correlation, *p* values < 0.05 were considered statistically significant, and *p* values < 0.1 were considered as trends. All computational procedure and statistical analyses were performed using R, version 4.2.1, with RStudio, version 2022.07.1.

## 3 Results

We analyzed data from three-hundred fourteen CU participants from the ALFA+ prospective cohort study, with complete CSF biomarkers and awareness measurements. Participant demographic characteristics are shown in Table 1. Two-hundred five (65.3%) individuals were classified as A-T-, eighty-six (27.4%) individuals as A+T-, and twenty-three (7.3%) individuals as A+T+. SCD was defined in eighty-seven (27.7%) individuals, and non-SCD in two-hundred twenty-seven (72.3%) individuals. Study partner’s SCD was defined in twelve (3.8%) individuals, and study partner’s non-SCD in three-hundred two (96.2%) individuals. SCD-Q total scores for My-Cognition and Their-Cognition were also provided in Table 1, along with descriptive characteristics about study partners.

**TABLE 1:** Participant characteristics

	Full sample (*n* = 314)	A-T- (*n* = 205)	A+T- (*n* = 86)	A+T+ (*n* = 23)
**Demographic**				
Age, mean (SD)	60.9 (4.73)	60.2 (4.53)	61.6 (4.93)	63.8 (4.42)
Female sex, *n* (%)	186 (59.2)	120 (58.5)	50 (58.1)	16 (69.6)
Years of education, mean (SD)	13.6 (3.55)	13.8 (3.46)	13.9 (3.59)	11.3 (3.47)
**Genetic**				
*APOE-*ε4 carriers, *n* (%)	173 (55.1)	90 (43.9)	70 (81.4)	13 (56.5)
**Biomarker**				
CSF Aβ42/40, mean (SD)	0.0744 (0.0191)	0.0863 (0.0087)	0.0534 (0.0109)	0.0463 (0.0110)
CSF p-tau181 (pg/mL), mean (SD)	15.5 (5.83)	13.9 (4.18)	15.6 (4.15)	29.6 (4.87)
CSF t-tau (pg/mL), mean (SD)	190 (60.9)	175 (47.6)	191 (45.2)	327 (44.8)
**Neuropsychological**				
MBT Objective performance, mean (SD)	66.1 (13)	66.7 (13)	66.2 (12)	59.9 (15.1)
MBT Subjective performance, mean (SD)	66.2 (15.5)	66.1 (15.6)	67.3 (15.1)	62.9 (16.3)
MBT Meta-memory performance, mean (SD)	−0.118 (11.3)	0.62 (11.7)	−1.11 (10.4)	−2.99 (11)
**Subjective cognitive decline**				
**SCD-Q my-cognition**				
SCD, *n* (%)	87 (27.7)	56 (27.3)	25 (29.1)	6 (26.1)
My-cognition, mean (SD)	4.16 (4.45)	3.98 (4.42)	4.24 (4.12)	5.43 (5.68)
**SCD-Q their-cognition**				
Study partner’s SCD, *n* (%)	12 (3.8)	8 (3.9)	4 (4.7)	0 (0)
Their-cognition, mean (SD)	1.51 (2.48)	1.42 (2.48)	1.70 (2.64)	1.70 (1.84)
**Psychiatric**				
HADS Anxiety, mean (SD)	3.60 (2.91)	3.51 (2.87)	3.56 (3.10)	4.61 (2.41)
HADS Depression, mean (SD)	1.71 (2.16)	1.68 (2.25)	1.59 (1.84)	2.48 (2.39)
**Study partner**				
Age, mean (SD)	57.5 (12.6)	57.5 (12.8)	57.0 (12.8)	59.6 (10)
Female sex, *n* (%)	174 (55.41)	115 (56.09)	47 (54.65)	12 (52.17)
**Relationship with the participant**				
Partner, *n* (%)	219 (69.75)	144 (70.24)	58 (67.44)	17 (73.91)
Son/Daughter, *n* (%)	14 (4.46)	8 (3.90)	4 (4.65)	2 (8.70)
Sibling, *n* (%)	21 (6.68)	17 (8.29)	3 (3.49)	1 (4.35)
Other, *n* (%)	60 (19.11)	36 (17.56)	21 (24.42)	3 (13.04)

The CSF biomarker cutoffs used to define AT groups were 0.071 for the CSF Aβ42/40 ratio, and 24 pg/mL for CSF p-tau181, which were previously validated in ALFA+ cohort study. Neuropsychological data: episodic memory was evaluated with the Memory Binding Test (MBT), scores presented for objective and subjective performance were scaled ranging from 0 to 100, and meta-memory was defined as the normalized discrepancy between objective and subjective performance. The presence of subjective cognitive decline (SCD) and study partner’s SCD was defined if the answer to this initial general question in the SCD-Q was affirmative: “*Do you perceive memory or cognitive difficulties?*” for the participant, and “*Do you perceive he/she has cognitive or memory difficulties?*” for the study partner. Total scores for My-Cognition and Their-Cognition accounted for an overview of the daily living instances in which cognitive changes were noticed in the last two years (i.e., higher scores indicated higher complaint). Psychiatric data: anxiety and depression were evaluated using the Hospital Anxiety and Depression Scale (HADS).

Participant-partner contingency by SCD status is shown in Table 2. Within non-SCD (*n* = 227), study partner’s SCD was defined in seven (3.01%) individuals (characterized as unaware decliners; only the study partner presented SCD), and study partner’s non-SCD was defined in two-hundred twenty (96.91%) individuals (characterized as controls; neither participant nor partner reported SCD). Within SCD (*n* = 87), study partner’s SCD was defined in five (5.75%) individuals (characterized as aware decliners; self-reported SCD confirmed by the study partner), and study partner’s non-SCD was defined in eighty-two (94.25%) individuals (characterized as SCD not confirmed by the study partner).

**TABLE 2:** Subjective cognitive decline (SCD) status by participant-partner parallel questionnaires.

SCD-Q My-Cognition	SCD-Q Their-Cognition		Total, *n* (%)
	Study partner’s non-SCD, n (%)	Study partner’s SCD, *n* (%)	
Non-SCD, *n* (%)	220 (70.06)	7 (2.23)	227 (72.29)
SCD, *n* (%)	82 (26.11)	5 (1.59)	87 (27.71)
Total, *n* (%)	302 (96.18)	12 (3.82)	314 (100)

SCD-Q My-Cognition in the rows, and SCD Their-Cognition in the columns, representing participant-partner contingency by SCD status, respectively. Data corresponding to the control group presented in cell [1, 1], data corresponding to the group of unaware decliners in cell [1, 2], data corresponding to SCD not confirmed by the study partner in cell [2, 1], and data corresponding to aware decliners in cell [2, 2].

### 3.1 Associations between meta-memory and CSF biomarkers

**FIGURE 1:**
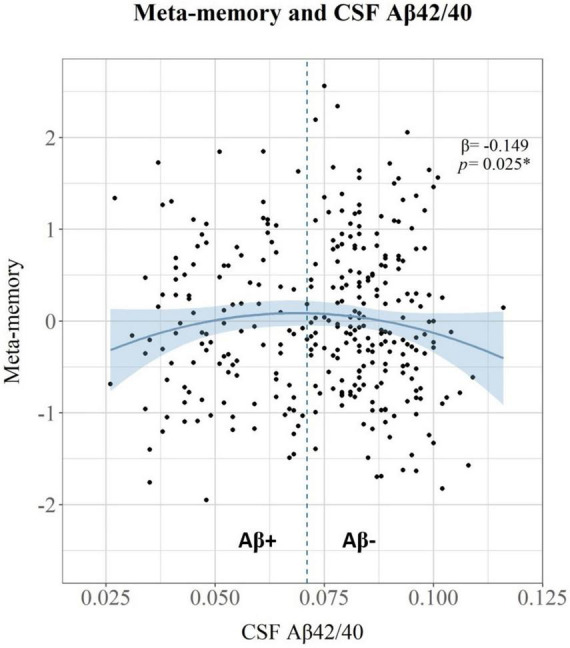
Scatter plot showing the relationship between CSF Aβ42/40 on the *X*-axis, and meta-memory standardized residual scores adjusted for demographic effects on the *Y*-axis. The vertical dashed line at *x* = 0.0071 represents the positivity threshold for CSF Aβ42/40. Standardized β coefficient and *p* value for the quadratic association between CSF Aβ42/40 and meta-memory standardized residuals are shown in the top-right side.

Meta-memory raw scores were regressed against the demographic data (considering sex, age, and education) to obtain meta-memory standardized residual scores adjusted for these effects. The relationship between meta-memory standardized residuals adjusted for demographics and CSF biomarkers was evaluated. We entered simultaneously in a multivariable regression model the following predictors: CSF p-tau181, CSF Aβ42/40, the quadratic term of CSF Aβ42/40, and the interaction between CSF p-tau181 and the quadratic term of CSF Aβ42/40 (Table 3). Meta-memory was significantly associated with the quadratic term of CSF Aβ42/40 (β = −0.149, *p* = 0.025), but not with CSF p-tau181. Figure 1 shows the relationship between CSF Aβ42/40 and meta-memory standardized residuals. These results show a non-linear pattern (inverted-U shape) with increased awareness of episodic memory (i.e., hypernosognosia) being associated with lower Aβ42/40 levels up to approximately the threshold of Aβ-positivity (CSF Aβ42/40 < 0.071), followed by lower awareness (i.e., anosognosia) being associated with lower Aβ42/40 levels, after surpassing the threshold of Aβ-positivity.

**TABLE 3:** Cerebrospinal fluid (CSF) biomarkers associated with meta-memory standardized residuals.

Predictors	std. β (95% CI)	*p* value
Intercept	0.116 (−0.046, 0.279)	0.160
CSF p-tau181	−0.003 (−0.153, 0.146)	0.967
CSF Aβ42/40	−0.046 (−0.173, 0.081)	0.474
CSF (Aβ42/40)^2^	−0.149 (−0.280, −0.019)	0.025*
CSF p-tau181 × (CSF Aβ42/40)^2^	0.057 (−0.029, 0.142)	0.194

Results presented are standardized β coefficients, 95% Confidence Interval (CI), and *p* values derived from multivariable regression models using CSF Aβ42/40, and CSF p-tau181 as predictors of meta-memory standardized residuals adjusted for demographic characteristics (sex, age, and education). The *p* values in bold indicate a significant association (* < 0.050).

### 3.2 Stratified analyses by SCD status

**FIGURE 2:**
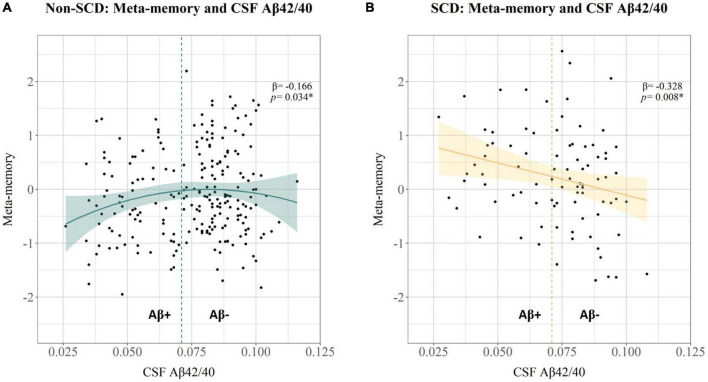
Scatter plots showing the relationship between CSF Aβ42/40 on the *X*-axis, and meta-memory standardized residual scores adjusted for demographic effects on the *Y*-axis, for **(A)** the subset of non-SCD, and **(B)** the subset of SCD. The vertical dashed lines at *x* = 0.0071 represent the positivity threshold for CSF Aβ42/40. Standardized β coefficient and *p* value for the quadratic **(A)** and linear **(B)** associations between CSF Aβ42/40 and meta-memory standardized residuals, respectively, are shown in the top-right side of each plot.

To further explore the role of SCD, the sample of participants was stratified according to SCD status. The relationship between meta-memory standardized residuals and CSF biomarkers was evaluated in both subsets (non-SCD and SCD). We entered simultaneously in two separate multivariable regression models the following predictors: CSF p-tau181, CSF Aβ42/40, the quadratic term of CSF Aβ42/40, and the interaction between CSF p-tau181 and the quadratic term of CSF Aβ42/40 (Table 4 and Figure 2). In the non-SCD subset, meta-memory was significantly associated with the quadratic term of CSF Aβ42/40 (β = −0.166, *p* = 0.034), but not with CSF p-tau181. In the subset with SCD, meta-memory was significantly associated with the linear term of CSF Aβ42/40 (β = −0.328, *p* = 0.008), but not with CSF p-tau181.

**TABLE 4:** Subjective cognitive decline (SCD) stratification: CSF biomarkers associated with meta-memory standardized residuals.

Predictors	Non-SCD		SCD	
	std. β (95% CI)	*p* value	std. β (95% CI)	*p* value
Intercept	0.118 (−0.073, 0.310)	0.225	0.145 (−0.160, 0.450)	0.348
CSF p-tau181	−0.007 (−0.187, 0.174)	0.942	−0.006 (−0.279, 0.268)	0.968
CSF Aβ42/40	0.075 (−0.074, 0.225)	0.322	−0.328 (−0.570, −0.087)	0.008*
CSF (Aβ42/40)^2^	−0.166 (−0.318, −0.013)	0.034*	−0.148 (−0.401, 0.105)	0.247
CSF p-tau181 x (CSF Aβ42/40)^2^	0.082 (−0.026, 0.191)	0.137	0.003 (−0.138, 0.144)	0.964

Sample was stratified by SCD-Q My-Cognition, non-SCD with *n* = 227, and SCD with *n* = 87. Results presented are standardized β coefficients, 95% Confidence Interval (CI), and *p* values derived from multivariable regression models using CSF Aβ42/40, and CSF p-tau181 as predictors of meta-memory standardized residuals adjusted for demographic characteristics (sex, age, and education). The *p* values in bold indicate a significant association (* < 0.050).

### 3.3 Incorporation of study partner’s SCD report

To further explore the contribution of dyadic SCD reports, in this section we considered exclusively the subset of participants with study partner’s SCD (3.82%). Within this subset, seven individuals (58 %) were defined as unaware decliners (non-SCD & study partner’s SCD), and five individuals (42 %) were defined as aware decliners (SCD & study partner’s SCD). For each group, meta-memory standardized residuals were evaluated using Spearman correlation matrices with CSF Aβ42/40, and CSF p-tau181. In the group of unaware decliners, meta-memory was significantly correlated with CSF p-tau181 (*r* = −0.857, *p* = 0.024), but not with CSF Aβ42/40 (*r* = 0.571, *p* = 0.200). Within the group of aware decliners, meta-memory was correlated at the trend level with CSF p-tau181 (*r* = −0.900, *p* = 0.083), but not with CSF Aβ42/40 (*r* = −0.500, *p* = 0.450).

### 3.4 Sensitivity analysis

The sensitivity analysis evaluated the effect of *APOE-*ε4 status and psychiatric symptoms in these associations. We incorporated *APOE-*ε4 status in a multivariable regression model (see Supplementary Table 1). While *APOE-*ε4 status was not associated with meta-memory, the effect of the quadratic term of CSF Aβ42/40 remained significant in the model (β = −0.151, *p* = 0.025). We incorporated anxiety and depression sub-scores from the HADS in multivariable regression models (see Supplementary Table 2). Although higher levels of anxiety were significantly associated with increased meta-memory (β = 0.131, *p* = 0.032), the effect of the quadratic term of CSF Aβ42/40 remained significant in the model (β = −0.142, *p* = 0.033). On the other hand, higher levels of depression were associated, now at the trend level, with increased meta-memory (β = 0.100, *p* = 0.082) while the effect of the quadratic term of CSF Aβ42/40 remained significant in the model (β = −0.134, *p* = 0.047).

## 4 Discussion

This cross-sectional observational research evaluated the association between core CSF AD biomarkers and the awareness of episodic memory performance, further exploring the role of SCD in these associations at the preclinical stage of the Alzheimer’s continuum. In the whole sample of participants, main results indicated that CSF Aβ42/40 was associated with meta-memory performance, following a non-linear pattern where higher Aβ burden was associated with both increased awareness (i.e. hypernosognosia) and decreased awareness (i.e. anosognosia) of episodic memory. This relationship showed an inverted U-shaped with the inflexion point approximately aligned with the Aβ-positivity cutoff. Therefore, these results are consistent with prior evidence suggesting that non-linear dynamic changes in meta-memory might be associated with Aβ-deposition in preclinical-AD (Vannini et al., 2017a; Gagliardi et al., 2020).

The present research hypothesized that, at very early stages in the preclinical Alzheimer’s continuum, two distinct types of metacognitive profiles at risk of AD-related decline could be delineated:

(I) Hypernosognosia: Individuals with a sub-estimation of actual memory performance, along with an increased awareness in relationship to AD-neuropathology. This increase in awareness is expected to anticipate gradual meta-cognitive decline with further disease progression.
(II) Anosognosia: Individuals with an over-estimation of actual memory performance, along with decreased awareness in relationship to AD-neuropathology. This decrease in awareness is expected to anticipate substantial meta-cognitive decline with further disease progression, and is, therefore, suggestive of greater risk of Alzheimer’s severity.

Our findings support the existence of these two groups, indicating that distinct meta-cognitive profiles may emerge in preclinical-AD. On the one hand, results in the non-SCD subset showed a non-linear association between Aβ and meta-memory, similar to the relationship found in the whole sample, with increased awareness up to Aβ-positivity, and decreased awareness beyond this threshold. On the other hand, results in the SCD subset showed a linear association between Aβ and meta-memory, with higher Aβ-pathology associated with increased awareness. These results suggested that while some individuals might notice subtle cognitive changes (SCD) associated with the entrance in the AD continuum (defined by the Aβ-positivity threshold), others may not be fully aware of these subtle changes (non-SCD). Although these groups differ in SCD status, both support meta-cognitive profile (I) hypernosognosia, showing sub-estimation of actual memory performance associated linearly and non-linearly, respectively, with Aβ-pathology.

Besides, we explored the contribution of the study partner’s SCD. On the one side, the group of unaware decliners, those individuals presenting only study partner’s SCD, revealed a distinct pathological link with meta-memory performance, showing that increased levels of CSF p-tau181 (i.e., higher pathology) correlated with lower meta-memory performance. On the other side, the group of aware decliners, those individuals presenting study partner’s SCD but now in agreement with self-reported SCD, revealed a similar link with meta-memory performance: showing that increased CSF p-tau181 was correlated, at the trend level, with decreased meta-memory. Despite the small sample size, these results rapidly suggested that the absence of SCD should not be taken as guarantee of cognitive stability. Indeed, both unaware and aware decliners exhibited a similar relationship between CSF p-tau181 and meta-memory performance. Although these groups also differ in SCD status, both support meta-cognitive profile (II) anosognosia, showing over-estimation of actual memory performance in linear relationship to higher tau-pathology.

In the present research, we proposed that both hypernosognosia and anosognosia, as inter-individual meta-cognitive profiles, can be identified, providing relevant information to enhance the characterization of the preclinical stage in the Alzheimer’s continuum. These meta-cognitive profiles were described with distinct methods used to define awareness of cognitive function (objective-subjective discrepancy, participant self-report, participant-partner discrepancy), capturing in turn, distinct features of the actual cognitive state of an individual. We showed that these features can be combined to enhance clinical characterization. To contextualize this observation, we acknowledge that the co-occurrence of SCD with low awareness, or vice versa, might seem contradictory. However, it serves to increase the true dimensionality, as it has already been documented in the literature. Indeed, previous studies have already informed that low awareness can coexist together with SCD in the same individual, in line with our findings showing that some individuals reported cognitive complaints but still over-estimated their actual performance, which in turn reveals under-estimation of deficit severity (Cacciamani et al., 2017, 2020, 2021). Reduced awareness into the degree of deficit severity, even in the presence of reports of cognitive decline, is already indicative of anosognosia, presented here as a negative discrepancy in objective-subjective performance. Following this same reasoning, the participant-partner discrepancy in SCD that defined the group of unaware decliners contributed to disentangle an over-estimation of actual memory performance in relation to higher levels of tau-pathology. These results suggested that some individuals might be overlooked with the use of actual diagnostic criteria simply because they do not report cognitive complaints. The present research is consistent with previous evidence showing that just self-reported SCD alone, might be insufficient information, but incorporating the study partner’s SCD could be necessary to distinguish signs of decline that could go otherwise unnoticed (Miebach et al., 2019; Nosheny et al., 2022).

In consistency with these findings, our group has previously suggested that the presence of the study partner’s SCD, irrespective of self-reported SCD, was associated with lower left posterior hippocampal GM volume. Moreover, we previously found that the group of unaware decliners displayed lower performance in free memory recall and increased GM volume in medial frontal and insular brain areas, suggesting subtle neuronal disruption in cognitive control and self-referential processing (Sánchez-Benavides et al., 2018a). Currently, anosognosia is not fully recognized in MCI diagnosis, and consequently, as a function of the resources available to the clinician and the strategy used to evaluate awareness, individuals with a meaningful deficit of anosognosia might not fit within MCI criteria (Albert et al., 2011). The assessment of SCD is an important part of MCI diagnosis, but the “liberal” use of SCD (*e.g.*, assuming that there is no possible lack of awareness prior to clinical onset) might contribute to misdiagnosis (Mitchell, 2008). Therefore, identifying a group at increased risk of AD-related impairment that might delay seeking for medical care is a matter of equity. In the next years, it is expected that effective treatments for AD will become available, and current research indicates that these treatments could be more effective at early stages in the AD continuum (Boxer and Sperling, 2023). Considering that decreased, but not increased awareness, reflects a greater risk of AD-related decline (Edmonds et al., 2014), improving our understanding of the pathological mechanisms behind anosognosia would provide useful methods and knowledge, from diagnosis to intervention, to close this equity gap.

We considered necessary to provide further explanation for the heterogeneous levels of awareness detected in preclinical-AD. Thus, we explored subthreshold symptoms of anxiety and depression, which are known to be possible manifestations associated with SCD and AD-progression (Jessen et al., 2014). Therefore, we modeled the relationship between CSF biomarkers, affective symptoms, and meta-memory as part of the sensitivity analysis. Higher anxiety was associated with increased awareness, and higher depression showed a trend in the same direction. The quadratic term of CSF Aβ42/40 remained significant in these models, indicating that affective symptoms partially overlapped with the effect of AD neuropathology in awareness, which is consistent with subtle manifestations emerging with underlying AD-progression (Donovan et al., 2018; Krell-Roesch et al., 2018). These results were in line with previous literature showing that subclinical levels of anxiety have been associated to a greater extent than those of depression with Aβ-pathology (Lewis et al., 2022).

Although non-linear changes in awareness across preclinical AD have already been suggested, we proposed that distinct levels of cognitive awareness shown by different individuals might be characterized considering inter-individual meta-cognitive profiles that can help to explain distinct intra-individual dynamics and corresponding trajectories. In the present research, we identified cross-sectionally these distinct profiles: while some individuals experience a heightened awareness of subtle cognitive decline (often leading them to report concerns and/or seek for medical advice), others can demonstrate a lack of insight across distinct dimensions of cognitive awareness (objective-subjective discrepancy in neuropsychological performance, and participant-partner discrepancy in SCD reports). Nevertheless, the rationale behind these distinct pathways to anosognosia is unknown. The literature shows that subtle meta-memory alterations in CU individuals have been associated with Aβ pathology in brain regions overlapping with areas involved in awareness and self-referential processing (Vannini et al., 2017a; Gagliardi et al., 2020). In amnestic MCI patients, greater anosognosia has been associated with reduced functional connectivity in cortical midline structures and reduced metabolism in the hippocampus and precuneus (Vannini et al., 2017b). Considering our results, it remains uncertain whether distinct these meta-cognitive profiles are fundamentally related to the progression of distinct AD neuropathology, as defined by independent contributions of Aβ and Tau pathologies, and/or related to patterns of pathological progression deviating from traditional schemes (Vogel et al., 2021; Collij et al., 2022).

Characterizing subtle meta-cognitive alterations might result challenging considering the underlying pathophysiological heterogeneity present in preclinical-AD cohorts and methodological differences across research settings (demographics, inclusion criteria, biomarker/cognitive measurements, etc.). These factors might contribute, to some extent, to explain the distinct associations between AD biomarkers and levels of awareness in preclinical-AD described in recent review (Cacciamani et al., 2021). The early anosognosic profile, unaware decliners, as defined by the participant-partner discrepancy in SCD, was quite infrequent in our sample (*n* = 7), but similar in sample size to the group of aware decliners (*n* = 5). Among participants presenting study partner’s SCD, some participants did not present awareness of cognitive decline, while other participants reported SCD in agreement with their partner (58–42% in our sample, respectively). Further development of methodology for defining this group at risk, might consider applying a cut-off in the objective-subjective discrepancy in neuropsychological performance to capture a larger group of individuals with similar performance characteristics, helping to understand better why some individuals might display a reduced awareness of cognitive function from very early stages. Clearly, longitudinal research needs to account for the characterization of this early anosognosic trajectory and its pathological correlates (in preparation).

A key feature of this research was to account for a sample of relatively young participants, with a mean age of 60 years old, showing a high contrast with most of studies in the field of preclinical-AD, with mean ages around 75. Moreover, the ALFA+ cohort study is mainly composed of early biomarker profiles in the Alzheimer’s continuum (A-T-/A+T-) and further enriched by risk factors (AD parental history, and *APOE-ε4* carriers). The ALFA+ study was designed as an observational prospective follow-up of cognitively healthy middle-aged volunteers at increased risk of AD dementia. Therefore, the report of SCD was obtained on request rather than inclusion criteria. The first and only study to date that has directly modeled meta-memory performance as a function of the non-linear effect of Aβ-PET in CU individuals was performed in the context of SCD samples. In the present study we replicated the non-linear association previously described by Gagliardi et al. (2020) between Aβ and meta-memory, but now considering CSF biomarkers and a broader spectrum of population (only the 27% of the present sample in the ALFA+ cohort presented SCD). Since CSF Aβ levels are expected to change slightly before amyloid PET imaging, using fluid biomarkers, the present research was focused at the very early stages of the preclinical Alzheimer’s continuum (Jack et al., 2013).

### 4.1 Limitations

The present research presented some limitations, as shown by the distribution of AT profiles, the levels of AD pathology were considered from modest to low. We acknowledge that only 23 (7.32%) individuals were classified as A+T+, currently falling within the profile of AD, and this might have obscured some latent relationships between the awareness of cognitive function and the levels of CSF p-tau181, which reflects greater symptom severity than CSF Aβ42/40 does with further disease progression. Other limitations relate to possible confounders, previous research identified that education levels were associated with meta-cognitive performance, but the ALFA+ cohort is mainly composed of highly educated individuals and our results did not yield any significant association with education. A group of interest, namely the unaware decliners, was also very limited in sample size, as previously stated, restricting the use of inferential statistics. Additionally, the report of study partner’s SCD might be influenced by several factors like age, sex, education, and the participant-informant relationship. Similarly, other relevant factors not accounted for in these analyses, such as personality traits related to mental stability (neuroticism, openness, etc.), together with cultural aspects, might contribute to explain distinct levels of awareness of cognitive function. Finally, we have used very sensitive cut-offs in the ALFA+ cohort study for the AT(N) classification system (Milà-Alomà et al., 2020). Therefore, we acknowledge that using more liberal cut-offs for classifying pathological status, or other methods such as PET imaging, might lead to distinct results.

## 5 Conclusion

This research suggested that distinct meta-cognitive profiles can be identified at the preclinical stage of the Alzheimer’s continuum. While most individuals might experience an increased awareness associated with the entrance in the AD continuum (i.e., hypernosognosia), some others might be already losing awareness (i.e., anosognosia). Reduced awareness of cognitive function at preclinical stages of AD might currently be overlooked, leading to the omission of some individuals at higher risk of cognitive decline who may not be considered in prevention studies. This research suggested that further characterization of these meta-cognitive profiles might enhance preclinical stage identification, providing insights into the likelihood of clinical progression from the very early stages in the Alzheimer’s continuum.

## Data availability statement

The data that support the findings of this study are available from the corresponding co-authors, OG-R and GS-B, upon reasonable request.

## Ethics statement

The studies involving humans were approved in the ALFA+ study (ALFA-FPM-0311) by the independent Ethics Committee “Parc de Salut Mar,” Barcelona, and registered at Clinicaltrials.gov (identifier: NCT02485730). The studies were conducted in accordance with the local legislation and institutional requirements. The participants provided their written informed consent to participate in this study.

## Author contributions

DL-M: Conceptualization, Data curation, Formal analysis, Investigation, Methodology, Software, Visualization, Writing – original draft, Writing – review and editing. MS-C: Funding acquisition, Resources, Writing – review and editing. MM-A: Writing – review and editing. JG: Funding acquisition, Resources, Writing – review and editing. CM: Project administration, Resources, Writing – review and editing. CQ-R: Resources, Writing – review and editing. GK: Resources, Writing – review and editing. HZ: Resources, Writing – review and editing. KB: Resources, Writing – review and editing. OG-R: Conceptualization, Funding acquisition, Investigation, Project administration, Resources, Supervision, Writing – review and editing, Formal analysis, Methodology, Validation, Visualization. GS-B: Funding acquisition, Investigation, Project administration, Resources, Supervision, Writing – review and editing, Conceptualization, Formal analysis, Methodology, Validation, Visualization.
