# Supplementary material for: Awareness of episodic memory and meta-cognitive profiles: associations with cerebrospinal fluid biomarkers at the preclinical stage of the Alzheimer’s continuum
Source: Front Aging Neurosci. 2024 May 30;16:1394460. doi: 10.3389/fnagi.2024.1394460 (PMC11169691; doi:10.3389/fnagi.2024.1394460)
Supplement: Supplementary file 1 [file Table_1.docx]

Supplementary Material

**Sensitivity analyses**

Sensitivity analyses evaluated the effect of *APOE-*ε4 status and psychiatric symptoms in these associations. We incorporated in a multivariable regression model *APOE-*ε4 status (**Supplementary Table 1**). While *APOE-*ε4 status was not associated with meta-memory, the effect of the quadratic term of CSF Aβ42/40 remained significant in the model (β= -0.151, *p*= 0.025). No other association with any other predictor was found.

| **Supplementary Table 1.** Sensitivity analysis adjusting for *APOE-*ε4 status: demographics and CSF biomarkers associated with meta-memory standardized residuals. | | |
| --- | --- | --- |
| **Predictors** | **std. β (95% CI)** | ***p* value** |
| Intercept | 0.137 (-0.082, 0.356) | 0.220 |
| CSF p-tau181 | -0.004 (-0.154, 0.146) | 0.958 |
| CSF Aβ42/40 | -0.054 (-0.192, 0.084) | 0.444 |
| CSF (Aβ42/40)^2^ | -0.151 (-0.282, -0.019) | **0.025*** |
| CSF p-tau181 x (CSF Aβ42/40)^2^ | 0.056 (-0.030, 0.142) | 0.200 |
| *APOE-ε4* status | -0.034 (-0.276, 0.209) | 0.784 |
| Results presented are standardized β coefficients, 95% Confidence Interval (CI), and p values derived from multivariable regression models using CSF Aβ42/40, and CSF p-tau181 as predictors of meta-memory standardized residuals adjusted for demographic characteristics (sex, age, and education). The values in bold indicate *p* < 0.100 ( · < 0.100, * < 0.050). | | |

The effect of subtle psychiatric symptoms: anxiety and depression, was evaluated in meta-memory performance. We incorporated in separate multivariable regression models anxiety and depression sub-scores from the HADS (**Supplementary Table 2**). While higher levels of anxiety were significantly associated with increased meta-memory (β= 0.131, *p*= 0.032), the effect of the quadratic term of CSF Aβ42/40 remained significant in the model (β= -0.142, *p*=0.033,). No other association with any other predictor was found. On the other hand, higher levels of depression were associated at the trend level with increased meta-memory (β= 0.100, *p*= 0.082) and the effect of the quadratic term of CSF Aβ42/40 remained significant in the model (β= -0.134, *p*= 0.047). No other association with any other predictor was found.

| **Supplementary Table 2.** Sensitivity analysis adjusting for psychiatric symptoms: CSF biomarkers and anxiety or depression (independent models), associated with meta-memory standardized residuals. | | | | | |
| --- | --- | --- | --- | --- | --- |
| HADS Anxiety | | | HADS Depression | | |
| **Predictors** | **std. β (95% CI)** | ***p* value** | **Predictors** | **std. β (95% CI)** | ***p* value** |
| Intercept | 0.115 (-0.047, 0.277) | 0.163 | Intercept | 0.105 (-0.058, 0.268) | 0.208 |
| CSF p-tau181 | 0.011 (-0.138, 0.161) | 0.88 | CSF p-tau181 | 0.003 (-0.147, 0.152) | 0.973 |
| CSF Aβ42/40 | -0.033 (-0.160, 0.094) | 0.61 | CSF Aβ42/40 | -0.035 (-0.163, 0.093) | 0.591 |
| CSF (Aβ42/40)^2^ | -0.142 (-0.272, -0.011) | **0.033*** | CSF (Aβ42/40)^2^ | -0.134 (-0.266, -0.002) | **0.047*** |
| CSF p-tau181 x (CSF Aβ42/40)^2^ | 0.046 (-0.039, 0.132) | 0.287 | CSF p-tau181 x (CSF Aβ42/40)^2^ | 0.051 (-0.035, 0.137) | 0.246 |
| HADS Anxiety | 0.131 (0.019, 0.242) | **0.022*** | HADS Depression | 0.100 (-0.013, 0.212) | **0.082·** |
| Results presented are standardized β coefficients, 95% Confidence Interval (CI), and p values derived from multivariable regression models using CSF Aβ42/40, and CSF p-tau181 as predictors of meta-memory standardized residuals adjusted for demographic characteristics (sex, age, and education). The values in bold indicate *p* < 0.100 ( · < 0.100, * < 0.050). | | | | | |
